# Pain Management for Animals Used in Science: Views of Scientists and Veterinarians in Canada

**DOI:** 10.3390/ani4030494

**Published:** 2014-08-01

**Authors:** Nicole Fenwick, Shannon E. G. Duffus, Gilly Griffin

**Affiliations:** Canadian Council on Animal Care (CCAC), 190 O’Connor St., Suite 800, Ottawa, ON, K2P 2R3, Canada; E-Mails: nfenwick@ccac.ca (N.F.); shannon.duffus@gmail.com (S.E.G.D.)

**Keywords:** analgesia, animal models, interview study, pain management, refinement

## Abstract

**Simple Summary:**

Veterinarians, veterinarian-scientists and scientists (all engaged in animal-based studies in Canada) were interviewed to explore the challenges and opportunities for laboratory animal pain management. Our broader aim was to contribute to further discussion of how pain can be minimized for animals used in science. Recognizing when animals are in pain continues to present a challenge, and there does not seem to be consensus on the signs of pain. Clarification of the interactions between scientific objectives and pain management are needed, as well as a stronger evidence base for pain management approaches. Detailed examination of pain management for individual invasive animal models in order to develop model-specific pain management protocols may be useful.

**Abstract:**

To explore the challenges and opportunities for pain management for animals used in research an interview study with 9 veterinarians, 3 veterinarian-scientists and 9 scientists, all engaged in animal-based studies in Canada, was carried out. Our broader aim was to contribute to further discussion of how pain can be minimized for animals used in science. Diverse views were identified regarding the ease of recognizing when animals are in pain and whether animals hide pain. Evidence of inconsistencies in pain management across laboratories, institutions and species were also identified. Clarification of the interactions between scientific objectives and pain management are needed, as well as a stronger evidence base for pain management approaches. Detailed examination of pain management for individual invasive animal models may be useful, and may support the development of model-specific pain management protocols.

## 1. Introduction

The possibility that animals may experience pain when used in science presents an ethical dilemma for both scientists and laboratory animal veterinarians. The dilemma applies both in cases where the pain is a direct consequence of the research (*i.e.*, where pain is the area under study), and in cases where pain is an indirect consequence. The globally accepted ethic of animal experimentation (based on the Three Rs of Russell and Burch [1]) requires that animal pain and distress be minimized (Refinement) [2]. However in some types of animal-based research, pain alleviation measures may be in conflict with scientific objectives. Therefore, concerns regarding the adequacy of pain management continue to be expressed in the scientific literature [3,4,5].

Some studies have aimed to quantify the prevalence of analgesic administration following surgery and/or other potentially painful procedures in a variety of laboratory animal species [6,7,8,9]. The studies concluded that the use of analgesia has increased over time, but the proportion of animals reported as receiving analgesics remains less than the proportion subjected to painful procedures, especially in the case of smaller species [6,7,8,9]. Similarly, a Canadian survey of analgesia-withholding found that over 12 months, 42,700 animals (approximately 1.9% of total national use) were used in invasive protocols that received animal ethics committee (AEC) approval to withhold analgesia [10].

Some studies have also identified reasons why analgesia may be withheld or not used. A United Kingdom (UK) survey of scientists and animal care staff found that although respondents had a broad awareness of when there was a potential for animal pain, their accuracy in detecting pain was complicated by a lack of pain indicators and the use of subjective criteria, which may contribute to under-use of analgesia [11]. In the United States (US), a roundtable discussion involving veterinarians, technicians and AEC members identified the lack of knowledge about techniques used to assess, monitor and treat pain as one of the main barriers to reduction of pain in laboratory animals [5]. In the Canadian survey on analgesia-withholding, reasons scientists withheld analgesia included: when analgesia was *proven* conclusively to interfere with experimental results; when analgesia *may* interfere with experimental results; and when pain was part of the phenomenon being studied [10].

To further explore and describe the challenges and opportunities for laboratory animal pain management we undertook a qualitative interview study with veterinarians, veterinarian-scientists and scientists (all engaged in animal-based studies in Canada). Our broader aim is to contribute to further discussion within the scientific community of how pain can be minimized for animals used in science.

## 2. Methods

A qualitative research approach was selected so that we could elicit and describe the opinions and perspectives of our participants. Using this approach meant that we did not attempt to comprehensively or statistically represent the views of all veterinarians, veterinarian-scientists or animal-based scientists [12]. In addition, our participants are involved in animal-based research in Canada, and therefore it is possible that some of their perspectives and experiences may not resonate with individuals in working in jurisdictions with different animal use oversight mechanisms and/or research environments. To inform our reporting of methodological details we consulted the “consolidated criteria for reporting qualitative research (COREQ)” checklist [13]. Twenty-one interviews were conducted between March and August 2012 (one additional interview was conducted but discarded due to poor quality of the audio recording). Participants included 9 laboratory animal veterinarians (who provided care for animals and had oversight of animal-based research), 3 veterinarian-scientists (who provided care for animals, had oversight of animal-based research and carried out animal-based research) and 9 scientists (who carried out animal-based research). Ethics approval was obtained from Institutional Review Board (IRB) Services (Aurora, ON, Canada) (ICF 111018).

### 2.1. Participant Selection

Purposive and snowball sampling methods [14] were used to recruit participants who were involved with the use of animals in potentially painful research at Canadian academic institutions. Our initial goal was to recruit participants from each province in Canada (four each from Ontario and Quebec, having the greatest concentration of research institutions and two from each other province). Therefore, an invitation to participate was distributed by email to a listserv of 218 Canadian AEC chairs asking them to distribute the invitation to relevant potential participants. Participant recommendations were also solicited from people known to have an interest in this topic and then invitations to participate were sent via email to the recommended individuals. Subsequently, the participant pool was expanded through referrals from other participants (snowball sampling [14]). We cannot report precisely on the response rate to all our requests for participants because the number of individuals who may have seen the listserv invitation but did not respond is not known. However, five individuals who were contacted directly declined to participate (1 declined outright and 4 initially agreed but then did not respond to subsequent attempts to contact them).

### 2.2. Participant Demographics

To collect demographic information participants were asked to complete a questionnaire in advance of the interview. Twelve participants were male and 9 were female; 17 participants were current or past members of AECs. Participants’ length of professional experience in animal-based science ranged from 5 to 40 years (see Table 1 for experience ranges). Collectively, participants worked in 7 Canadian provinces and 12 different academic institutions. All these institutions had staff veterinarians except for one.

**Table 1:** Participants’ length of professional experience in animal-based science.

	Early Career (<10 years)		Experienced (>10 years)	
	Number of Participants	Years of Experience (Range)	Number of Participants	Years of Experience (Range)
scientists	4	6–9	5	18–40
veterinarians	3	5–10	6	13–22
veterinarian-scientists	1	8	2	20

The 9 scientists and 3 veterinarian-scientists described their research areas as: autoimmune disease, neurobiology, cancer, neuroendocrinology, neurology, neuropharmacology, pain, pharmacology, spinal cord injury, stroke recovery and veterinary medicine. The 9 scientists reported having experience with the following rodent species: mice, rats and gerbils. Participants with veterinary degrees (9 veterinarians, 3 veterinarian-scientists) collectively had experience with a wider range of species including: amphibians, birds, cats, cattle, chinchillas, dogs, ferrets, fish, goats, horses, invertebrates, non-human primates, pigs, sheep, rabbits, reptiles, rodents (including mice, rats and gerbils) and various wild species.

### 2.3. Interviews

Participants were provided with the study description and consent and confidentiality agreements prior to being interviewed. In the semi-structured interviews participants were asked a series of open-ended questions that had been pilot-tested with the assistance of two veterinarians and one animal-based scientist who were not participants. Interviews, which lasted 1 to 2 h, were conducted face-to-face (7) or by telephone (14) and all were audio-recorded. At the start of each interview, the participant was given a verbal summary of the study and, for face-to-face interviews, asked to sign the consent form. For telephone interviews a signed consent form was obtained in advance. A single investigator carried out all the interviews (SD).

Participants were asked to discuss: (i) procedures and/or animal models they are involved with that may cause pain; (ii) standard drugs and regimes they use to manage pain; (iii) the role of handling and husbandry in pain management; (iv) how an animal in pain is identified; (v) training on recognizing animal pain that is provided at their institution; (vi) how animal pain is monitored; and (vii) what improvements are needed for the management of animal pain. At the end of the interview participants were also asked if there was anything that was not talked about that they considered important. During interviews the term “pain” was used as an umbrella term for stimuli and experiences ranging from a pin prick to severe pain. Participants were not asked to provide a definition of animal pain and the term “animal” was used throughout interviews without necessarily specifying species.

The open-ended style of the interviews allowed participants to respond to each question for as long as they wished and encouraged dialogue, therefore questions were not always delivered in the same order; in many cases participants brought up topics before the question was asked. This format also provided the interviewer with the flexibility to ask follow-up questions, prompted by points brought up by the participant.

### 2.4. Data Analysis

Interview recordings were transcribed verbatim and numbers were assigned to each participant to anonymize the transcripts. Each transcript was read several times and analysis began with the identification of key concepts and ideas from the text, a process known as coding [15]. Codes were developed both from responses to interview questions and participants’ comments that could not be directly related to questions. To develop the coding scheme, two coders (NF and SD) coded the same five interviews and discussed and further refined the coding scheme together. One coder (NF) coded the remaining interviews. The coded sections were then grouped into themes as relationships between codes emerged. In general, our analysis did not attempt to compare the views of one participant group to another as this would not be appropriate with our small, non-statistically representative sample and because not every participant commented on every topic.

To illustrate the research findings, quotations from participants are used, selected on the basis of how well they reflected a given idea and to use quotes from all participants. The quotes have been presented verbatim, although they have been edited to remove interjections (e.g., “um”, “uh”) and to add punctuation. To provide context for the quotes, they are each ascribed to the participants’ role and level of experience (Table 1) and, for veterinarian-scientists and scientists, their self-described research area.

## 3. Results and Discussion

### 3.1. Recognizing when Animals are in Pain

#### 3.1.1. Views on Whether Animals are Experiencing Pain Due to Research Use

Some participants readily acknowledged that some animals will unavoidably experience some type of pain as a consequence of scientific use:

“*in terms of animals with, you know, moderate to severe unalleviated pain, I can’t think of a project where that would be, you know, the expected outcome. I think there are individual cases, in situations where if people are not effectively assessing or monitoring an animal, that there would be a case where it would experience some pain that was unalleviated*”

(early career veterinarian)

“*it would be unrealistic to suggest that we could ever not have animals in pain or distress when* *we use them in research*”

(experienced veterinarian-scientist studying pharmacology)

Some participants felt that pain in animal-based research is currently well-managed or that anything greater than momentary pain does not occur widely in animal research:

“*most of the time we are able to find something to give some analgesia to the animal ... we don’t have really a big problem with that because if it’s not possible to give a systemic drug, we at least are able to do local anesthesia*”

(experienced veterinarian)

“*I tend to believe, and I hope it’s not just wishful thinking, but I tend to believe that, or I want to believe that the pain in our procedures is quite minimal*”

(early career scientist studying neurobiology speaking of his/her own research)

“*if it doesn’t hurt, why are we worried?*”

(experienced pain scientist)

When discussing their local experiences, some study participants perceive that animal pain is well-managed and/or minimal. If this perception was widespread in the animal-based research community then it would be unnecessary to try and draw further attention to unalleviated animal pain and remedy the challenges to animal pain management. In contrast, Canadian and international animal use statistics annually document that animal use does occur at the highest levels of severity, where there is potential for significant pain and distress [16,17,18].

#### 3.1.2. Ease of Recognizing Pain

Participants held diverse and contradictory views regarding how easily pain can be recognized in laboratory animals. Five of the scientists and one veterinarian expressed confidence that they could readily recognize pain in their own research animals, in part due their knowledge of the individual animals under their care:

*“either the animals are super, are fine, after two hours all this is normal or they’re not and when they’re not, they’re still in a corner, they’re not moving, they’re not reacting*”

(experienced neuroscientist explaining how s/he determines whether to provide analgesia)

“*they* [animals] *never seem to hold a grudge against me, you know, the day after a surgery, if, you know, they haven’t had buprenorphine* [analgesic drug] *and all that and they seem like they’re fine the day after, they don’t seem to be upset with me, they come back up to the cage and be interested in playing with me and stuff like normal*”

(early career scientist studying stroke)

In contrast, about half of the participants (including veterinarians, veterinarian-scientists and scientists) expressed some uncertainty about their ability to recognize pain and/or felt that recognizing animal pain was difficult:

“*I don’t think he’s feeling pain but, you know I’m not, I don’t know, I’m not a veterinarian, I don’t have that expertise*”

(early career neuroscientist recounting the condition of a rodent)

“*I have no idea if they* [the animals] *are in pain or not, you know, my veterinary training is telling me that probably pain is there but I don’t know because I cannot see it, they don’t show, either I don’t know which behavioral signs will reveal pain or they don’t have, they don’t show the behavioral signs that will reveal pain, so, that’s a problem*”

(experienced veterinarian)

Some of the participants who believed that recognizing pain was difficult further commented specifically on the difficulties of recognizing chronic pain:

“*I’m not sure that I would be able to, truly identify just looking at the animal, if it’s kind of a bearable pain*”

(experienced veterinarian discussing diagnosis of chronic pain)

“*the whole issue of chronic pain is very underdiagnosed maybe not diagnosed at all*”

(early career veterinarian)

Differing views on whether animals hide their pain were also expressed with some participants believing that animals do hide pain:

“*it’sevolutionarily very important for them to hide pain*”

(experienced veterinarian-scientist who studies pharmacology)

“*the rats are hiding the level of pain, so when you look, subjectively, to* [the] *degree of discomfort and pain, no one was able to detect the rats that were injected with MIA* [painful adjuvant] *and the rats that were injected with saline*”

(experienced veterinarian-scientist who studies pain)

Other participants did not support the idea that animals would (or could) hide their pain:

“*some people have told me that they think that animals hide pain, that’s nonsense*”

(experienced pain scientist)

“*we expect that rats will come and run to greet us, if the rat’s in pain, they don’t do that at all*”

(early career stroke scientist)

“*a rat that’s not comfortable will let you know, he or it, I guess, is not comfortable*”

(early career neuroendocrinologist)

“*Oh, they’re showing signs, we’re just not smart enough to see it*”

(early career veterinarian)

To recognize pain, some participants explained how they relied, in part, on their prior experience with animals:

“*I’ve worked with them long enough that, that I feel that I can, I can tell, there’s certain signs in their facial features, the fur, their behavior, that I’m pretty good, I think, at noticing when something’s not right but, again I think it comes with a lot of experience too*”

(early career scientist studying neuroendocrinology)

“*a number of the students that we deal with really don’t have any experience with animals of any nature, and so that, in and of itself, creates a very significant challenge*”

(experienced veterinarian-scientist who studies pharmacology)

In contrast, just one participant discounted the importance of experience:

“*there’s no way to tell, you know by sitting in front of an individual animal and I just don’t buy the fact that there are people that know because they have so much experience*”

(experienced pain scientist)

The finding that recognizing animal pain is perceived as challenging is supported by other literature (as discussed in introduction) [4,5,11,19]. It also occurs in other areas of animal use. For example, a 2012 survey of Australian veterinarians’ attitudes to providing post-operative analgesia for companion dogs found that 1/5 of respondents did not have confidence in their knowledge of pain and 42% had difficulties recognizing when dogs were experiencing pain [20].

Some participants felt that their experience with animals and pain recognition was helpful for them when making decisions about animal pain management. In contrast, some published research contradicts this view. For example, one study found that experienced observers were no better at detecting pain in rabbits than naive observers [21]. In addition, a review of the usefulness of ‘observer ratings’ of animal behavior found that although observers with close knowledge of the individual animal are more skilled at observing their behavior, they have a tendency to see positive indicators of welfare in animals that are under their own care [22]. This may contribute to explaining why some participants expressed confidence in their ability to recognize when their own animals were experiencing pain.

#### 3.1.3. Ways of Identifying Pain

Participants reported using a variety of signs to aid in pain recognition including observations of animal behavior and physical appearance, and physiological measurements (e.g., weight, temperature, respiration rate). Participants described using the following behavioral observations: absence of grooming, audible vocalization, decreased social interaction, facial expressions, hunched posture, impaired mobility, lack of nest building (for mice), lethargy, not drinking, over-grooming, place preference (*i.e.*, preference for certain location), reduced appetite, self-mutilation and writhing (for rodents). The following physical signs were described: appearance of masses or bodily secretions, beady eyes (for rodents), coat appearance, dehydration, ocular porphyrin staining (for rodents) and swelling. However, participants also acknowledged these signs can be unreliable and not necessarily indicative of pain:

“*we don’t rely on only one clinical sign to tell us something about the animal, and not every clinical sign is associated with pain, it could be just simply distress or stress”*

(experienced veterinarian-scientist studying pharmacology)

“*we could have something better on there* [pain-scoring checklist]*for specific pain-related things, these are kinda’ just general appearance and mobility things that we have on there*”

(early career neuroscientist discussing his/her institution’s pain-scoring checklist)

“*with postoperative pain, with any pain stimulus that lasts over about 30 min, you don’t get any behavior at all. That’s the problem we have*”

(experienced pain scientist)

The use of pain-scoring checklists to monitor animals and make decisions about pain alleviation was also discussed and differing views about their usefulness emerged:

“*we’ve demanded them from all our, for all protocols submitted to … our Animal Research Ethics Board. So these humane intervention checkpoints will identify scores which an intervention is provided and they have to, the PIs* [scientists], *have to define that intervention*”

(experienced veterinarian-scientist who studies pharmacology)

“*my first reflex is just not to have to score pain, but just to assume pain is there and to treat it. There’s always the question of, if you wait for it, to score it then, by the time you score it, the animal has been in pain for, a few hours”*

(experienced veterinarian)

*“we spent a lot of work, probably for the last 25 years on* [a]* subjective pain scale … I think it’s a loss of time, is not reproducible, is not repeatable and, even not sensitive”*

(experienced veterinarian-scientist who studies pain)

About one third of participants commented on how analogies to human experience of similar procedures are used as a way to identify when animals may experience pain:

“*when in doubt we basically assume it, it’s going to hurt, if we think it would hurt us*”

(early career veterinarian)

“post-operative pain only lasts a few days and, even if we don’t know that in mice and rats, we assume it because we know in humans post-operative pain only lasts a couple of days”

(experienced pain scientist)

Guidance documents for using laboratory animals recommend the use of checklists and the identification of clinical signs as a way to determine whether an animal is experiencing pain and distress, and as a tool to determine *a priori* when an experiment should be terminated [23,24]. However, other ways of determining animal pain are now being described in the literature. Examinations of rodent facial expressions and pain-scoring based on expressions has also shown that standard ways of detecting pain can be inaccurate [25,26,27]. Other research into rodent pain behaviors has shown that signs of pain are more subtle than previously understood [28], and has led to the development of automated pain-detection methods [29,30]. These findings combined with the difficulties of recognizing pain as shown in the literature and acknowledged by some participants in this study, suggest that experience with animals may not be sufficient for reliable pain detection.

#### 3.1.4. Types of Pain

Many participants commented on the difficulties associated with using the term ‘pain’ as an umbrella term for all painful stimuli and experiences. Some participants felt it would be preferable to correlate the level of concern over pain with its intensity and the animals’ ability to “*cope*”:

“*what is more important, is it the pain that we are able to cope with or the pain that we’re not able to cope with? Therefore, if you have a chronic pain that you are not able to manage, to cope with you, you will be needing treatment for the pain but if you are able to live with that pain and … seem to be quite good with it, I mean, is it truly needed to treat that pain*?”

(experienced veterinarian using an analogy to the experience of people dealing with a painful condition)

“*this comes down to a matter of intensity which is something that’s lost I think in these discussions, people say the word pain and they’re free to, by using the same word, they’re free to equalize everything from, you know, the pain of trigeminal neuralgia to a bee sting*”

(experienced pain scientist)

These participants felt that this lack of nuance resulted in animal pain being addressed with, inappropriately, ‘one-size-fits-all’ solutions. They felt that different types of pain should lead to both different levels of concern for animal welfare and different pain management actions. For example, an early career scientist working in stroke research described that the analgesia schedule required to be used at his/her institution was appropriate for a major surgery but not for less invasive procedures. S/he explained that his/her experimental procedures involved creating, “*a small cut and it is on the animal’s head but it’s not severe in the same way as other surgeries but we’ve got, we were initially lumped in*” with more invasive procedures. Similarly, another experienced pain scientist commented, “*animal care recommendations don’t differentiate between kinds of pain*.”

These comments about different types and intensities of pain suggest that when assessing pain it is important to determine what the animal is actually experiencing (rather than simply assessing, for example, what procedure is performed on the animal) [19,31].

### 3.2. Pain Management

#### 3.2.1. Approaches to Pain Management

Participants described using both pharmacological and non-pharmacological approaches to alleviating animal pain. Pharmacological approaches included using opioid analgesics, non-steroidal anti-inflammatory drugs (NSAIDs), local and topical anesthetics, as well as general anesthetics during surgery. Many participants reported that they gave groups of animals standardized pain treatment as well as treated animals individually. However, two participants (one veterinarian and one scientist) said they do not provide individualized treatment.

When asked about non-pharmacological pain alleviation strategies, participants described measures related to improving animal comfort, such as softer bedding, lowered lighting and using heat pads or lamps. They also reported lowering water bottles and placing food on the cage floor so that animals in pain would not have to stand and reach for food or water. Changes to food were also mentioned, such as improving nutritional composition, softening food and emphasizing rehydration. Animal handling was mentioned specifically in relation to rodents, including minimizing handling and use of special containers for handling mice, and increasing handling and interaction for rats (although participants did not elaborate on why this may alleviate pain).

Some participants mentioned they use preventative measures such as developing skill in surgical techniques, and ensuring proper techniques are used to administer drugs:

“*it’s quite important to choose the right site, not too close to tail base, then there will be more severe inflammation*”

(experienced scientist working on autoimmune diseases describing how injection sites are selected)

Some veterinarians and veterinarian-scientists mentioned defining experimental endpoints as a way to limit and hence manage animal pain. In addition, a few veterinarians also mentioned that occasionally pain alleviation is achieved by presenting scientists with the choice of either providing analgesia or having animal care staff humanely kill an animal prior to conclusion of the experiment.

Participants were asked about what they saw as opportunities to improve pain management for research animals. Some identified technology and the use of automated animal monitoring systems for detecting animals in pain. One participant suggested improving animal nutrition. Others suggested changes related to the use of pharmaceuticals including: improvements to drug formulations so that they are longer acting with longer dosing intervals; use of a greater variety of drugs and drug combinations; routine use of pre-operative medications; and mandatory administration of analgesia and local anesthesia during surgeries. 

Other opportunities related to services provided by animal care professionals, such as the use of more animal care staff and centralized animal care and use facilities:

“*a couple* [of] *really well trained animal techs who could do surgeries for PIs* [scientists], *as opposed to the PI or the graduate student doing the surgery, to ensure that you have better outcomes*”

(experienced veterinarian-scientist who studies pharmacology)

The participants in this study described using both pharmacological and non-pharmacological approaches to managing animal pain. Using non-pharmacological measures when analgesics must be withheld is an approach that has been advocated for the refinement of animal models in pain research [4], however, changing the “standard of care” and expectations for the analgesics so that they are universally required (similar to use of anesthetics in surgery) has also been proposed [3,32].

#### 3.2.2. Inconsistencies in Pain Management

Example of inconsistencies in pain management practices across institutions, laboratories and species were mentioned by participants:

“*our lab was kinda split between* [institution 1] *and* [institution 2] *at the time, we did a studyin* [institution 1] *where we had somewhat more lax requirements of what we had to use for analgesia, and we tested, using just a topical, or local anesthetic after surgery, one injection of buprenorphine and then the three injections of buprenorphine used here* [institution 2] *and we saw some effect on the severity of the stroke, in terms of behavioral deficits, as well as an increased mortality in animals that were receiving buprenorphine injections*”

(early career stroke scientist describing the different analgesia protocols between two institutions conducting the same research)

“*we did that* [give analgesics]*at* [previous institution], *we don’t do it at* [present institution], *even though I guess we should, and I wouldn’t have to ask anyone, I should just do it myself*”

(early career scientist studying neuroendocrinology)

Inconsistencies in pain management strategies and resources provided for different species was raised by a few veterinarian participants:

“[in a] *large animal operating room, the simple budget of one animal might be, might be 5–10 thousand dollars and in that 5–10 thousand dollars we’ve got trained animal health technicians, we’ve got controlled drugs and we’re able to do that, whereas in a mouse project, if they’re doing 100 mouse surgeries with a graduate student and the budget’s smaller, they don’t have that, so, so it’s a huge issue*”

(early career veterinarian)

Two veterinarians (one experienced, one early career) commented that pain management options for fish are lacking. Another early career veterinarian commented on how the physical size of the animals affects what can be done for them:

“*it’s very challenging if not impossible to give an epidural to a mouse or a rat and you can do that for a dog, I think there are some biases because of circumstance that the larger, some of the larger species are probably get more state of the art, if that’s what you wanna’ term it, analgesia then the rodents do. However I wouldn’t, I don’t feel in most cases like the rodents are getting insufficient analgesia*.”

Inconsistencies in analgesia were also attributed by an early career veterinarian (different from above) to difficulties obtaining certain drugs:

“*they’re not doing it because it takes more effort, it takes more time, some of the drugs are controlled so they have to get their controlled drug license at this institution*.”

A further example of inconsistency in pain management also emerged when some participants commented on the use of carprofen analgesia in rodent drinking water:

“*I’ve seen tons of SOPs and we have started using it ourselves, putting carprofen in the drinking water of mice prior to doing ear notching and tail amputations, as an attempt at pre-operative anesthesia- or analgesia preemptive analgesia. However we do this knowing there isn’t any information out there as to how efficacious it is, how stable it is in water, people have just looked at it anecdotally and a few like, they see some benefit to it and also suggest that they probably aren’t doing any harm by doing it*”

(early career veterinarian)

“*we are adamant, you do not put analgesics in the water, you look at the animal and you administer because what happens when you do water is, they’re painful they don’t drink, they’re, ‘stuff’s in the water’, no student comes by to look and to us that’s not* [an]*acceptable form of postoperative monitoring*”

(early career veterinarian, different from above)

Although our sample was small, a number of inconsistencies in pain management were identified. These inconsistencies have the potential to impact research results as well as the welfare of the animals. However, typically this information is not included in the methods sections of papers that arise from the work, as has been reported in studies that aimed to quantify the prevalence of analgesic administration [6,7,8,9]. Use of standardized checklists for reporting methodological details of animal-based research may assist in standardizing this information and decreasing inconsistencies in practice [33].

#### 3.2.3. Pain Management Knowledge

Some scientists described the pain management protocols they used in their research in great detail and complexity with reference to drug mechanisms and pain processes. Others described following the standard or pre-existing protocols at their institution, or the recommendations of the institutional veterinarian:

“*when you enter a lab and it’s a protocol they’ve had in place for years, you just kind of accept it and follow it*”

(early career neuroscientist)

“*the procedure that I walked into at* [institution], *I guess I couldn’t really tell you why they chose that*”

(early career neurobiology scientist commenting on his/her analgesic regime)

This type of situation was also referenced by an early career veterinarian who spoke of pain alleviation protocols as being “*inherited*.”

Some veterinarians would prefer that scientists had more knowledge of pain management:

“*anesthesia and analgesia are not the same thing and people confuse them so just because you’re unconscious does not mean you’re free of pain*”

(early career veterinarian commenting on what s/he perceived as lack of awareness in some scientists)

“*I think it’s important for them* [scientists]*to understand the basis for the decisions, and why we make them, and I think if they can understand that they can also understand how some of their actions in surgery might be problematic for an animal in terms of causing excessive amounts of trauma and the inflammation that comes along with it*”

(early career veterinarian, different from above)

“*in the university, when you’re going to work with a PhD that has no experience in medicine* [they] *will be very surprised when you tell them that the animal will receive five or seven, five or six different analgesics, you have to explain a lot*”

(experienced veterinarian compared working in a university to a research hospital with medical doctors, who have familiarity with complex analgesic protocols)

Some scientists observed that there is limited evidence-based information to assist in making pain management choices, especially for specific animal models:

“*veterinarians don’t even have it* [evidence-based medicine] *because there’s simply no evidence base, there’re not enough experiments being done, so that there would be any data, you know, to argue about*”

(experienced pain scientist comparing the human medicine knowledge base to veterinary medicine)

“*there’s a lot of research about, you know, how to manage pain in general and also with all the analgesics, like how effective are these analgesics for different kinds of pain, how long do they last, what kind of doses are effective however, when you start looking into more, specific models … even just looking at stroke in general, not even the* [specific stroke]*model that we use but, analgesia and stroke, how are these affecting each other, it starts to become a lot more sparse*”

(early career scientist studying stroke)

However, some participants felt that research to advance animal pain management knowledge is hampered by lack of resources and/or perceived importance of the work:

“*I think not lots of people participate in these experiments and they need the resource and also what maybe some people will think, it’s not so significant a contribution to the science*”

(experienced scientist, working on autoimmune diseases)

“no one’s gonna’ do it, no one’s gonna’ pay anybody to do it”

(experienced pain scientist commenting on possible research that could be done to improve pain management for the animal model s/he uses)

“*we all presume that there’s pain, I suppose, it’s just that, without any evidence of it, it’s hard for people to, it’s hard for the issue to raise up to the level of priority that it probably deserves*”

(experienced pain scientist, different from above)

Participants were in general agreement that there is a lack of scientifically proven information on how to manage animal pain and an absence of resources available to address it. The lack of evidence-based information gives people using animals little option but to make decisions about pain management based on inadequate knowledge. Lack of pain management knowledge, and more specifically lack of scientists’ knowledge of pain management were also identified as a barrier to the assessment and treatment of animal pain in a roundtable discussion of laboratory animal professionals (including veterinarians) [5]. It may be useful to clarify what scientists need to know about pain management, and whether it is sufficient for them to collaborate with the institutional veterinarian to devise pain alleviation strategies.

### 3.3. Pain Management and Research Objectives

Over half of the scientist participants (but no veterinarians or veterinarian-scientists) expressed concern that using analgesia may interfere with their experiments:

“*analgesic drugs are powerful drugs that can affect a lot of these processes, so then we get into a situation of where we’re using drugs that might interfere*”

(experienced stroke scientist commenting on the effect of analgesic drugs in stroke recovery)

“*we spend a lot of work controlling all that stuff* [experimental variables], *now you want me to add a variable? Now that’s not good science*”

(experienced pain scientist)

One early career scientist studying neuroendocrinology described delaying administration of analgesia due to concern that it would cause excess bleeding:

“*I don’t want that* [analgesia]*to interfere with my study whatsoever so what I do is I give it kinda’ half-way through the surgery so it kicks in a little bit later or maybe right after surgery as well and then, I mean, I understand the fact that, you know, the animal will wake up and have a huge headache but it’ll have to wait just a little bit until the, my drug will kick in rather than waking up bleeding with no headaches*.”

Other scientists and veterinarian-scientists were less concerned about pain control interfering with their research or introducing additional variability:

“*I’m a fan, a proponent of variability because if you can see a difference in a system where you know there’s a fair bit of biological variability, then I would suggest that whatever intervention, or whatever it is you are doing, that caused that difference between a control and a treatment group … I’d be more comfortable that that’s a real, a real outcome*”

(experienced veterinarian-scientist who studies pharmacology)

“*if there is an effect of the analgesic on the experimental results … it’s an error that we take and accept and it’s something that is embedded in all our data, so we may limit the generalization from our data to one lab to another lab but that’s typical, so, and besides that, I don’t see any limitations … I worry more about whose doing, running the experiment, it’s always a different undergraduate and the data never match, that’s more error than giving morphine to the rat*”

(experienced neuroscientist)

Veterinarians in this study questioned the effect of variability from pain control on experimental outcomes:

“*there’s no model that’s perfect. We’re willing to live with the limitations of a model, so why not accept things, that pain control is one of the limitations. That we say, ‘okay, well, that’s* [a]*fact of life’ you know. There are things we cannot do with animals and this is one of* [those]*things, we just have to live with it*”

(experienced veterinarian)

“*no one’s ever come back to me and said, ‘oh, I could identify those two animals that we treated with analgesia or increased analgesia’ … on their data*”

(early career veterinarian)

Approximately half of study participants (including veterinarians, veterinarian-scientists and scientists) described how unalleviated pain can interfere with research:

“*if they’re feeling pain they’re probably not going to do the tasks that we’re hoping to get them to do, since these tasks involve their injured paw … I would imagine if they’re in pain they’re not gonna’ want to, they either won’t do it or, I guess results could be skewed in, you know maybe making it look worse than it actually, their deficit might look worse than it actually is, that kind of thing, so yeah, I think it would definitely have an effect*”

(early career neuroscientist who uses behavioral tests in his/her research)

“when an animal is in pain, it will induce interference anyway in the process you are studying and this is well known, you have a lot of change induced by the perception of pain and that could affect not only the neuronal system but the hormonal system”

(experienced veterinarian-scientist who studies pain)

Some veterinarians commented on how withholding of analgesia should be justified to AECs:

“*when I started my practice, it was me who had to prove to them that the analgesic I was prescribing will not affect the study, now it’s the contrary, it’s them* [scientists]*who have to prove* [to] *the animal care committee* [AEC] *that their, the analgesic, any analgesic will have an impact on their study. So if they say, ‘analgesia needs to be withheld’, then they will have to provide papers that really say and really show*”

(experienced veterinarian)

“*they* [scientists] *maybe quote some paper that they wrote so I’d say can you send me a copy of that and I’ll look it over. We do have, we have had occasion where someone has said we think that this could be impactful, and so we, have said well what we’d like is for you to go ahead and use it* [analgesia] *and then compare that data to previously collected data and see if you can document variance in that data*”

(early career veterinarian)

“*we get a justification for, that this drug can interfere, but we rarely ask the researchers to explain if stress or pain itself can interfere with the, with the project and, also, we tend to forget that there are so many other factors that influence a model and sometimes we kind of stick on this one and forget all the other ones*”

(experienced veterinarian, different from above)

In this study, scientist participants raised concerns about pain management interfering with research results, while the veterinarian participants were less concerned. However, participants of all types also commented on how animal pain can impact research results. Assessment of the impact of pain control on research results and the impact of untreated pain on results both suffer from a lack of evidence/information. Efforts to clarify “*what it means to* ‘*affect the model*’” are needed [3] (p. 4) and [19,34].

### 3.4. Communication, Professional Relationships and Pain Management

Communication, especially between scientists and institutional veterinarians, emerged as an important component of animal pain management. Some veterinarians felt it was important that scientists perceived that they (the veterinarians) understand research, and are not only focused on the animals:

“*if you come at them* [scientists] *with solely the animal welfare side of things … you know, whether it’s pain or distress, environmental enrichment, analgesics, anesthetic, whatever, they tend to either glaze over or dig their heels in. When you come at them with a balanced approach of, you know, ‘I’m concerned about your, the robustness of your research model and, you know, by the way, it also ends up, it results in better animal welfare too if we were to do this’, then they’re far more receptive because they see that you’re actually thinking about their research*”

(experienced veterinarian)

“[my role is] *being an advocate for the animal*… [but I] *also play a role as a collaborator and a facilitator for a researchers, but a researcher’s perspective is often different from my perspective*”

(early career veterinarian)

Some scientists commented on how it was useful for them to pro-actively communicate with their institutional veterinarian:

“*we communicated* [with] *each other and discussed the procedure, so I think it’s quite, it’s quite helpful for them* [veterinarians] *to understand why we have to do this and also why we should not give the mice any analgesia*”

(experienced scientist studying autoimmune diseases)

“*for my purposes, and if the vet tells me that it’s* [a pain treatment] *more appropriate, I’m gonna’ go with her judgment, of course and my animals look great*”

(experienced neuroscientist describing trusting the expertise of their institution’s veterinarian)

Many veterinarians spoke about relying on their past experience with the individuals conducting the research when it came to checking compliance with analgesia administration:

“*we tend to know also the team who is working, so depending on the team we can have, additional monitoring of the animal if we feel that we’re not certain that they’re giving the analgesic properly*”

(experienced veterinarian)

“*there’s less involvement, obviously with the more experienced labs … we kind of leave it up to them, to let us know if it’s working or not*”

(early career veterinarian)

A few veterinarians (but no scientists) also described acting more assertively to mitigate pain. For example, an experienced veterinarian attributed the success s/he had in making changes to institutional analgesic routines, “*partly because I push a lot*,” while an early career veterinarian explained how s/he responded sometimes in situations where a scientist has been reluctant to provide supportive treatment to a sick animal:

“*you can’t warm it up, you can’t give it fluids, you can’t give it analgesics, then we’re gonna’ say okay we’re gonna’ kill it*.”

The importance of professional communication also emerged when participants spoke about other workers in research laboratories. For example, an early career veterinarian expressed concern with the turnover in laboratory personnel and the lack of communication that can occur:

“*in the rush to get a lot of work done and people, you know, summer students or grad students coming in and out of labs, there’s not really a lot of communication, everyone’s busy so they don’t necessarily discuss the fine, fine details of, you know, detecting pain in animals, what’s indicative of, you know appropriate pain management or not*.”

This participant also linked the success of pain alleviation to communication, worrying that steps described in an animal care protocol may not be implemented if there is a lack of communication. An early career scientist in neurobiology described how poor communication could also affect research objectives:

“*sometimes the* [animal care] *staff and what they want done can interfere with what needs to be done in the lab and so, sometimes you have to come to some kind of agreement as to, okay what drugs are we gonna’ use that manages pain enough but also doesn’t interfere with the studies that we’re trying to do*.”

Participants in this study described the usefulness of communication between the professionals engaged in animal-based research. Building on this strength, workshops and more formalized collaborations could seek to resolve questions regarding the respective roles of scientists and veterinarians in animal pain management, an approach that has also been proposed to improve professional communications about laboratory animal environments [35].

## 4. Conclusions

This study aimed to explore and describe the challenges and opportunities for pain management for animals used in science and, through this, contribute to discussions of how pain can be minimized. Previous research [6,7,8,9] has shown that that the use of analgesia for pain management of animals used in science has increased over time, but the proportion of animals reported as receiving analgesics remains less than the proportion subjected to painful procedures. Other survey and workshop studies identified some reasons why analgesia may be withheld or not used, such as lack of pain indicators, lack of knowledge about techniques used to assess, monitor and treat pain and when analgesia is proven or believed to interfere with experimental results [5,10,11]. The interview methodology of the present study has elaborated on these reasons and provided additional possible reasons for the gap between animal pain and pain management.

When speaking of their local experiences some participants in this study perceived that animal pain is well-managed and/or minimal, in contrast to concerns regarding the overall adequacy of pain management in animal-based science expressed in other literature [3,4,5] and animal use statistics that document animal use at high severity levels [16,17,18]. Similar to other studies for example, [5,11,28], we also found that recognizing when, and to what degree, animals are in pain continues to present challenges, in part because there does not seem to be consensus on the signs of pain.

A number of inconsistencies in pain management practices across institutions, laboratories and species that have the potential to impact research results and animal welfare were described by participants. However, typically this type of information is not included in the methods sections of papers that arise from the work, as has been reported in studies that aimed to quantify the prevalence of analgesic administration [6,7,8,9].

Participants were in general agreement that there is a lack of scientifically proven information on how to manage animal pain and an absence of resources available to address it, similar to the findings of other studies [5,10,11,28]. This suggests that clarification of the interactions between scientific objectives and pain management is needed, as well as a stronger evidence base for pain management approaches, as has also been proposed by other authors [28,36]. Animal pain management may be best addressed by discipline and/or model-specific research, considering the vastness of different conditions and circumstances of each research area. Detailed examinations of existing pain management protocols for individual animal models leading to development of standardized model-specific pain protocols may be a useful approach. Similarly, a review of behavior measurements of pain in rodents concluded that assessment of chronic pain likely needs to be procedure and species specific [28]. Model-specific protocols may be readily adopted by scientists, as it emerged that some scientists in this study willingly following established protocols at their institution.
